# Microbial Beta Glucosidase Enzymes: Recent Advances in Biomass Conversation for Biofuels Application

**DOI:** 10.3390/biom9060220

**Published:** 2019-06-06

**Authors:** Neha Srivastava, Rishabh Rathour, Sonam Jha, Karan Pandey, Manish Srivastava, Vijay Kumar Thakur, Rakesh Singh Sengar, Vijai K. Gupta, Pranab Behari Mazumder, Ahamad Faiz Khan, Pradeep Kumar Mishra

**Affiliations:** 1 Department of Chemical Engineering and Technology, IIT (BHU), Varanasi 221005, India; karanpandey.che17@itbhu.ac.in; 2 Department of Bioengineering, Integral University, Lucknow 226026, India; rishi.rathour21@gmail.com (R.R.); faizkhan@iul.ac.in (A.F.K.); 3 Department of Botany, Banaras Hindu University, Varanasi 221005, India; sonamjha222111@gmail.com; 4 Department of Physics and Astrophysics, University of Delhi, Delhi 110007, India; 84.srivastava@gmail.com; 5 Enhanced Composites and Structures Center, School of Aerospace, Transport and Manufacturing, Cranfield University, Bedfordshire MK43 0AL, UK; 6 Department of Agriculture Biotechnology, College of Agriculture, Sardar Vallabhbhai Patel, University of Agriculture and Technology, Meerut 250110, U.P., India; sengar065@gmail.com; 7 Department of Chemistry and Biotechnology, ERA Chair of Green Chemistry, Tallinn University of Technology, 12618 Tallinn, Estonia; 8 Department of Biotechnology, Assam University, Silchar 788011, India; pbmazumder65@gmail.com

**Keywords:** lignocellulosic biomass, cellulase enzyme, β-glucosidase, enzymatic hydrolysis

## Abstract

The biomass to biofuels production process is green, sustainable, and an advanced technique to resolve the current environmental issues generated from fossil fuels. The production of biofuels from biomass is an enzyme mediated process, wherein β-glucosidase (BGL) enzymes play a key role in biomass hydrolysis by producing monomeric sugars from cellulose-based oligosaccharides. However, the production and availability of these enzymes realize their major role to increase the overall production cost of biomass to biofuels production technology. Therefore, the present review is focused on evaluating the production and efficiency of β-glucosidase enzymes in the bioconversion of cellulosic biomass for biofuel production at an industrial scale, providing its mechanism and classification. The application of BGL enzymes in the biomass conversion process has been discussed along with the recent developments and existing issues. Moreover, the production and development of microbial BGL enzymes have been explained in detail, along with the recent advancements made in the field. Finally, current hurdles and future suggestions have been provided for the future developments. This review is likely to set a benchmark in the area of cost effective BGL enzyme production, specifically in the biorefinery area.

## 1. Introduction

The continuous and increasing usage of fossil fuels has led to their depletion. Moreover, it has introduced various environmental hazards in the form of pollutants [1]. Bioenergy has been declared by the International Energy Agency (IEA) as the highest source of growth, at ~30% in the renewable consumption sector over the period of 2018–2023 [2]. In the year 2017, solar, hydropower, wind, and bioenergy covered ~25% of the power demand [3] around the world. In spite of having multiple renewable energy sources, consistent energy production and even supply are not ensured, because of climate dependency [4]. In this context, biomass degradation and their conversion in to biofuels seems an impressive and promising option, as cellulosic biomass is abundantly available and it is renewable as well [5,6,7,8]. Lignocellulosic biomasses are present in most of a plant’s cell walls, and hence it marks its presence in a plentiful amount [4]. Examples of such lignocellulosic biomasses are waste materials like forest residues, and agricultural, municipal, and industrial activities. Additionally, they can also be grown as energy crops that do not compete with food crops [9,10]. In general, the lignocellulosic biomass is comprised of two polysaccharides—cellulose, a homo-polysaccharide, and hemicelluloses, a hetero-polysaccharide. In addition, an aromatic hydrocarbon lignin is also present, along with a smaller portion of ash, proteins, and pectin [4]. Cellulose is a high molecular weight linear polymer composed of β-glucose (5000–10,000 units) and linked by β-1,4-glycosidic bonds [11]. Moreover, cellulose is highly crystalline in nature, and that makes it challenging to convert into monomeric sugars through the hydrolysis process [12]. Hemicellulose contains approximately 150 repeating monosaccharide (C5 and C6 sugar) units, and the type of monomeric sugars present therein varies depending on the types of materials [11]. In general, before the hydrolysis of a biomass, pretreatment is required so as to break the lignin present in the outermost layer of the biomass. The pretreatment of lignocellulosic biomasses can be done by following physical, chemical, and biological methods using chemicals or enzymes. Nevertheless, these treatment methods may result in the partial or complete removal of the lignin, and release the free structure of cellulose and hemicellulose, which can be further converted in to sugars by enzymes, and subsequently in to fuels, following fermentation using various types of microorganisms [13]. 

The physical methods used for the pretreatment of the lignocellulosic biomass mainly perform the structural disruption of the lignocelluloses, but require a high energy input such as extrusion [14,15] or cavitation [16,17]. On the other hand, chemical methods are most commonly used for the pretreatment of lignocellulosic biomass through alkali and acidic treatment [18,19]. Although the chemical method is effective, the high cost of the chemical consumption makes this method non-economical for a pilot scale, whereas biological pretreatment via microorganisms is sustainable and environment friendly. After lignin removal through the pretreatment process, the enzymatic bioconversion of free cellulose and hemicellulose yields reducing sugars. The enzymatic hydrolysis of the pretreated biomass requires the cellulolytic enzymes to break the polymeric structure of the cellulose and hemicellulose. Although multiple enzymes such as cellulase, hemicellulase, xylanase, ligninase, and pectinase actively participate in the enzymatic conversion of biomass, cellulase is the most important enzyme, because of its efficiency to perform the complete hydrolysis of cellulose into sugar [20]. Furthermore, cellulase is a system of three different enzymes, namely, exoglucanase, endoglucanase, and beta-glucosidase (BGL), and these enzymes act synergistically for the hydrolysis of cellulose [21,22]. All of these three enzymes perform distinct functions in the complete hydrolysis of cellulose. Endoglucanases randomly act over the crystalline structure of cellulose and cleave the linear chains of glucose, which results in shorter chains giving two new chain ends [23]. These two exposed ends then become available for the action of exoglucanases, which extricate cellobiose and some glucose [23]. Ultimately, the final role is played by β-glucosidases (BGL) for the complete degradation of cellulose, which breaks cellobiose and cello-oligosaccharides into glucose molecules [24,25,26,27]. Thereafter, β-glucosidase is the final enzyme in lignocelluloses degradation, which decides the rate of the total conversion of lignocelluloses into glucose [20,28,29,30,31,32].

As a result of the significant role of β-glucosidase in the biofuel industry, its production is needed so as to enhance and match up with the current demand of industries. For the production of cellulases, *Trichoderma reesei* has been widely used, but the yield of β-glucosidases from it is very poor [33]. It has been reported that most of the β-glucosidases produced from *T. reesei* were found to attach to the cell wall during fungal growth, causing the secretion of β-glucosidase of a low quantity into the medium [34,35]. Hence, the extraction of the β-glucosidase becomes a tedious task, and therefore the production of β-glucosidases is insufficient [34]. Although both the bacterial and fungal microbial strains are well reported for BGL production, the genus Aspergillus of fungi such as *Aspergillus niger* and *Aspergillus Phoenicus* are reported to give a higher yield of the β-glucosidases enzyme [36]. The earlier existing commercial β-glucosidase enzyme Novozyme SP188 was produced from *A. niger*, on which higher concentrations of glucose showed deleterious effects [37]. Sorensen et al. in their study described a novel species, *Aspergillus saccharolyticus*, that was able to produce an even higher titer value of β-glucosidases than *A. niger*, and can also substitute the commercial production of BGL [38]. In addition, some bacterial strains have been reported as potential β-glucosidase producers, such as *Bacillus subtilis* [39] and *Acidothermus cellulolyticus* [40], which produce more thermostable BGL compared with fungi, but are slow producers. In a recent study, Chen et al. described the cloning of the BGL gene from *Bacillus licheniformis* into *Escherichia coli*, and the production of the β-glucosidase enzyme with45.44 U/mLactivity [41]. Furthermore, it is also well documented that the activity of β-glucosidases gets inhibited because of the feedback inhibition of the final product glucose [20]. This loophole has led to an increased number of findings about glucose tolerant and glucose stimulated β-glucosidases, dating back the last 20 years [42]. In a study, Tiwari et al. worked on *B. subtilis* RA10 for the production of the thermostable β-glucosidase, which could efficiently convert cellulosic biomass into fermentable sugar [43]. Researchers also tried to increase the β-glucosidases production using various strategies. The enhanced production of β-glucosidases may be achieved by co-culturing *Trichodermareesei* with some other fungi, resulting in the proliferation of the enzyme efficiency of cellulose hydrolysis [44,45]. At the same time, recombinant DNA technology offers an alluring option for the cost cutting of the process by developing recombinant *T. reesei*, which can produce an absolute saccharifying enzyme in an ideal amount, containing β-glucosidases [46]. In addition, the β-glucosidase activity may be increased by the heterologous expression of β-glucosidase from other fungi in *T.reesei*, such as *Neosartorya fischeri* [47], *Aspergillus aculeatus* [48,49], and Periconia sp. [50]. Still, no recombinant strain of *T. reesei* is available that could ideally produce all of the components of cellulase, regardless of insight, knowledge, and multiple genetic efforts. 

In view of the above facts, the present review evaluates the production and efficiency of the β-glucosidase enzyme in the bioconversion of the cellulosic biomass for the biofuel production process at an industrial scale. The significance of BGL in biomass conversion has been discussed along with the recent developments and existing issues. The production and development of the microbial BGL enzyme have been also explained in detail, along with the recent advancements made in this field. At last, the current hurdles and future suggestions have been provided for the further developments.

## 2. Industrial Importance of β-Glucosidase in Biofuels

**Figure 1:**
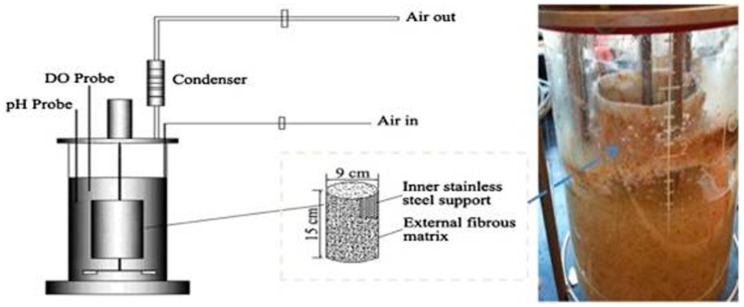
Higher β-1,4-glucosidase production by *Aspergillus niger* grown on wheat bran and glycerol was obtained in a rotating fibrous bed bioreactor (RFBB), because of better morphology control and mass transfer (adopted with permission from the authors of [59]).

Beta-glucosidase is a dual character enzyme that incorporates both the synthesis and degradation of the glycosidic bond, and this attribute of β-glucosidase makes it an enzyme with enormous potential from an industrial point of view [51,52]. The current scenario of raising the global energy demand day by day, and the increasing burden on fossil fuels, have necessitated biofuels production at a large scale so as to replace fossil fuels. The process of cellulosic biofuel production includes the breakdown of lignocellulosic biomass into sugar, followed by biofuel production through the fermentation process [20,52,53]. BGL is the key enzyme that ultimately converts cellobiose and cellooligosaccharide into a monomeric unit of glucose [54,55,56]. However, because of the insufficient BGL production, it becomes a rate limiting step of biofuel production technology [57,58]. Figure 1 represents the production process of the BGL enzyme at an industrial scale, using the fungal microorganism *A. niger* [59].

It has been noticed that because of the low efficiency of β-glucosidase and the unavailability of the potential BGL producer microorganism, the total conversion rate of cellulose to sugar is usually low. Moreover, the accumulation of cellobiose inhibits the other two enzymes of the cellulase complex, exoglucanase and endoglucanase [60,61]. The rate of hydrolysis, inhibitors, and stability, along with the product inhibition and thermal instability, are some of the major key factors that are needed in order to focus on achieving a higher conversion rate using BGL [58]. For example, a slow rate of hydrolysis and product inhibition have been noticed as the rate limiting step [62,63], while these bottlenecks can be overcome well by use the of [64,65] thermostable β-glucosidase, which has been documented in several studies [66,67]. Moreover, various research has been also performed, primarily focused on the heterologous expression and enzymatic cocktails, resulting in novel enzyme mixtures [68,69]. In a study, Chen et al. [70] worked on *Pichia pastoris*, which was expressing β-glucosidase encoding cDNA segregated from *Neocallimastix patriciarum*, a buffalo rumen fungus. This engineered enzyme showed better saccharification than the commercially available Novozym 188. In another study, Lee et al. modified *Saccharomyces cerevisiae* by the expression of the β-glucosidase and cellodextrin transporter from *Neurospora crassa*, and concluded the concomitant saccharification and fermentation that leads to a cost reduction [71]. In a recent work on the recombinant *E. coli*, Ferreira et al. [72] designed a new model for the cost effective production of β-glucosidase, and noticed the BGL had a yield of 15 g/L, making the final production cost ~37 US$/kg. 

For achieving the goal of economic biofuel production, many researchers have been working on different fungal and bacterial strains so as to obtain an effective cellulolytic fungal or bacterial β-glucosidase [73]. Liu et al. [74] reported on the increased hydrolysis of sugarcane bagasse with the help of BGL produced from *Anoxybacillus flavithermus* subsp.*yunnanensis* E13^T^. Yan et al., in their study, performed the hydrolysis of soybean isoflavones through the BGL obtained from *Aspergillus terreus* [75]. Liu et al. worked on *Aspergillus fumigatus* Z5, which gave a thermostable β-glucosidase, which was active even at an elevated temperature, and also, when added to the lignocellulosic biomass, it resulted in the removal of phenolic compounds, and hence it may be used for the degradation of polyphenols [76]. 

The use of thermostable and thermophilic β-glucosidase in cellulose hydrolysis is of particular interest, as a higher temperature of the process would not hinder the activity of BGL, and it also favors the hydrolysis process of the cellulose [77]. Multiple studies are present that describe the thermophilic and thermostable β-glucosidase. Dashtban and Qin isolated the thermostable BGL gene from Periconia sp., and inserted it into *T. reesei*, and hence the produced BGL showed an optimum activity at 60 °C [50]. Another study by Tiwari et al. involved *Bacillus subtilis* RA10 that produced thermostable BGL with 78% activity at 80 °C [43]. Zhang et al. isolated a thermophilic β-glcosidase from *Thermotoga naphthophila* RUK-10 and used it with cellulase for the hydrolysis of untreated corn straw, and observed an increase of 93.5% in the conversion rate from cellulose to glucose [77]. In a recent study, Fusco et al. synthetically produced a gene Dtur_0462 coding or β-glucosidase from *Dictyoglomus turgidum* and expressed in a strain of *Escherichia coli*, which resulted in the production of a thermostable β-glucosidase DturβGlu. The maximum activity of the produced β-glucosidase was observed at 80 °C, and it was able to exhibit 70% activity after the incubation of 2 h at 70 °C [78]. It was also described by Yeoman et al. that *Sclerotium glucanicum* [79] and *Aspergillus pheonicis* [80] mesophilic fungi grown at 24–27 °C produces β-glucosidase with an optimum temperature and stability of about 60–75 °C, and hence these fungi may be useful in order to produce thermophilic BGL at a normal temperature [81].

The co-culturing of different fungi for a higher yield of β-glucosidase has also been the focus of researchers [82,83,84,85]. Hu et. al. worked with *A. niger* and *Aspergillus oryzae*, along with some other strains like *Magnaporthe grisea* or *Phanerochaete chrysosporium.* The co-culturing of these strains displayed an improved production of enzymes, and the highest β-glucosidase activity was found for *A. oryzae* with *P. chrysosporium* [86]. *Trichoderma virdiae*, which produces the most commonly used cellulase enzyme, has a measurable β-glucosidase activity, and therefore the incorporation of thermo-tolerant BGL into cellulase preparation may give value addition effects, and hence an increased sugar concentration [31]. In a recent study, Zhao et al. combined the benefits of co-culturing and genetic modification by using the recombinant *T. reesei* mixed with the *A. niger* culture. This approach resulted in the most powerful cellulase with the highest enzymatic hydrolysis giving a yield of 89.35%, and for 1g of glucose production it needed the lowest input of cellulase (i.e., 25.18 filter paper unit FPU) [85]. Mallerman et al. worked on *Flammulina velutipes* CFK 3111, a white rot fungus, and observed the maximal β-glucosidase production of 1.6 U/mLwith a glucose production of ~10 g/L [87]. In a study, Jongmin et al. isolated *Aspergillus* sp.YDJ216, which produced two β-glucosidases, BGL1 and BGL2, and out of these two, BGL1 exhibited the maximum activity (953.2 U/mg) [88]. Abdella et al. worked on *A. niger*, and found the maximum BGL production at the repeated batch mode in the rotating fibrous bed bioreactor (RFBB) of about 1.78 U/mL/day [59]. In all of the above-mentioned studies on the BGL production, the best result was observed from the co-culturing of the recombinant *T. reesei* with *A. niger*, which has the potential of being used as a commercial producer of β-glucosidase [85].

Apart from biofuel production, β-glucosidase plays an important role in the beverage industry [24,32,57,89,90]. In wine production, BGL helps in the removal of the aromatic compound from the precursors of the glucosides present in fruit juices and musts [32,57], in the flavoring of tea [91], and fruit juice [90]. While working on the flavor and aroma enhancement of white muscat wine, Gonzalez-Pombo et al. [92] used *Issatchenkia terricola* for the isolation of β-glucosidase, which improved the flavor of the wine. Apart from beverages, the role of β-glucosidase is very fascinating in foods, especially those made from soy [93]. Glycosidic isoflavones, which are present in soy, are mainly daidzin, genistin, and glycitin [52]. These are largely inactive glycosides and need the activity of β-glucosidase to get them to convert into aglycones, namely daidzein, genistein, and glycitein [93]. Fermentation with a lactobacillus, producing β-glucosidase, or by treatment with β-glucosidase, resulted in a significant increase of aglycon in soy milk [94]. In addition, BGL synthesized a number of β-glucosides, abioflavanoid molecules that bond to glucose and are considered a subtype of glycosidase in plants. Additionally, because of its involvement in fundamental biological processes, multiple reports on its potential applications have been documented by the researchers, such as the hydrolysis of glucosyl ceramides in mammals and humans [95,96], the formation of glycoconjugates that play role in the defense mechanism of plants and microbes [24]. Beta-glucosidase acts on the precursors of glucosides found in fruits, and helps in the removal of aromatic compounds [97]. Because of this property, BGL is a crucial enzyme in the flavor industry. By performing reverse hydrolysis, β-glucosidase may also be used to synthesize the surfactant o-alkyl-glucoside, which may perform biological degradation, and also in food industry as a detergent [33]. Waste paper is currently a major environmental pollutant, and recycling it will give two-fold benefits, by reducing the consumption of forest wood and reducing landfill pollution [53]. There are two methods for recycling paper waste, either by the chemical or the enzymatic method, and because chemicals are environmentally hazardous, the use of enzymes like cellulase, β-glucsoidase, and hemicellulase is recommended [98,99,100,101]. Table 1 summarizes the numerous studies on the production of the BGL enzyme using the microbial process, following different physiological conditions, with the potential for biofuel application.

## 3. Classification of β-Glucosidase

Beta-glucosidases, a group of hydrolytic enzymes that are commonly present in fungi, plants, and bacteria, and the BGL obtained from them, share identical structures and sequences [52,122,123]. In general, β-glucosidases have been classified according to two methods of classification [20,52]. In the first system, the classification is based on the specificity towards the substrate, whereas in the second system, the nucleotide sequence identity is considered [20,42,52]. In the classification on the basis of the substrate specificity, β-glucosidases are subdivided into three groups—(1) aryl-β-glucosidase, which cleaves preferably aryl-glucosides; (2) cellobiases, which plays a role in the conversion of cellobiose, a disaccharide into glucose; and (3) glucosidases, which are found most commonly and have an extensive substrate specific activity over a wide range of substrates [124,125]. The above-cited second system of classification is widely accepted, and is based on both the identity of nucleotide sequence, as well as the structural similarity of the enzyme [126]. This system of classification keeps those enzymes in a single family, which shows similarity in sequences, and also has conserved sequence motifs [52,127]. It was also observed that near the active site of the enzymes, highly conserved amino acids were present in form of clusters [20,128]. The advantage of this kind of classification is that it enables the study of the evolutionary relationship, the structural features, and the mechanism of catalysis of these enzymes [20]. According to this classification, currently (as of June 2019), 164 families of glycosyl hydrolases (GH) exist, which can be accessed through the website of Carbohydrate Active enZYme (CAZY) [129]. The β-glucosidases are kept mainly in GH1 and GH3 families [52,64]. There are about 62 β-glucosidases present in the GH1 family, which are mainly obtained from archea, plants, and mammals, while the GH3 family is mainly comprised of about 44 β-glucosidase originated from bacteria, mold, and yeast [20,26,31]. GH families are also divided into clans, and those families with an identical catalytic domain and conserved amino acid domain that are proposed to share a common ancestry and mechanism of catalysis are placed in same clan. Most of the families, along with the families containing β-glcosidases, such as GH1, GH5, and GH30, are grouped in clan GH-A [52]. For the characterization and determination of the structures, the sequence-based classification is significant, but for determining the structure of the unknown and undefined glucosidases, the substrate specificity to the aglycone moiety is the most significant, and sometimes the only method of determination [20].

Glycosyl hydrolases families have been classified into their families with defined structures by the International Union of Biochemistry and Molecular Biology (IUBMB) [126,130]. Classification based on substrate specificity is not very informative, because there are multiple substrates that cannot be determined for an individual enzyme. However, classification on the basis of the structural features may provide structural information about the other members of the same family, with the help of bioinformatics tools [52].

Currently, the available classification systems seem more convenient, and hence are widely used, but some of the important factors are not considered in them, for example glucose tolerance and glucose-stimulation [42]. In one of the study, Cao et al. [65] categorized β-glucosidase on the basis of how glucose affecting the catalytic activity of β-glucosidase and this functional classification further categorized β-glucosidases into four classes [42], namely: (I) strong inhibition in low concentrations of glucose, (II) glucose tolerant, (III) stimulation under low concentrations of glucose and inhibition under the high glucose concentration, (IV) no inhibition at a high glucose concentration.

The β-glucosidases of class-I shows strong inhibition in low glucose concentrations, and these enzymes have avalue of Ki<0.1M for glucose [42]. This class of β-glucosidases includes the BGL of the GH3 family [131,132], some β-glucosidases of family GH1 [113,133], and those β-glucosidase, which are yet to be allocated into CAZY groups [134,135]. The β-glucosidase of class II are tolerant to glucose, and have a value of Ki>0.1M for glucose, and most of the characterized glucose tolerant β-glucosidases belong to the GH1 family, while having just one GH3 β-glucosidase obtained from *Mucor circinelloides*, as reported by Huang et al. [136]. The β-glucosidases belonging to class III show stimulation at low glucose concentrations, and the high glucose concentration inhibits their activity. However, compared with the absence of glucose, various glucose concentrations increase the activity of these enzymes [42]. The GH1 family includes this group of β-glucosidases, with two BGLs from the GH3 family. Class IV β-glucosidases do not get inhibited at higher concentrations of glucose, and they always exhibit a greater enzymatic activity at higher glucose concentrations than the activity in the absence of glucose [42]. In a study by Chan et al., it was found that β-glucosidases retained their 93% and 43% catalytic activity towards cellobiose at 10M and 15M concentrations of glucose, respectively [137].

The β-glucosidases show variation in their structure, but the overall catalytic domain of each GH family is identical [26]. Families belonging to Clan GH-A (GH1, GH5, and GH30) contain an identical (β/α)_8_-barrel, and two conserved carboxylic acid residues are also present at their active site [26,138]. In addition, the active site of the GH3 enzymes consist of two domain structures of the (β/α)_8_-barrel, along with having six stranded β-sheet and three α-helices sandwiched on either side [139]. In between (β/α)_8_ and (α/β)_6_, the active site is situated in theGH3 enzymes [26]. Most of the enzymes of the GH9 family are endoglycosidases, and a few are verified as β-glucosidase, which contain (α/α)_6-_barrel structures [140,141].

## 4. Catalytic Mechanism of β-Glucosidase Enzyme

**Figure 2:**
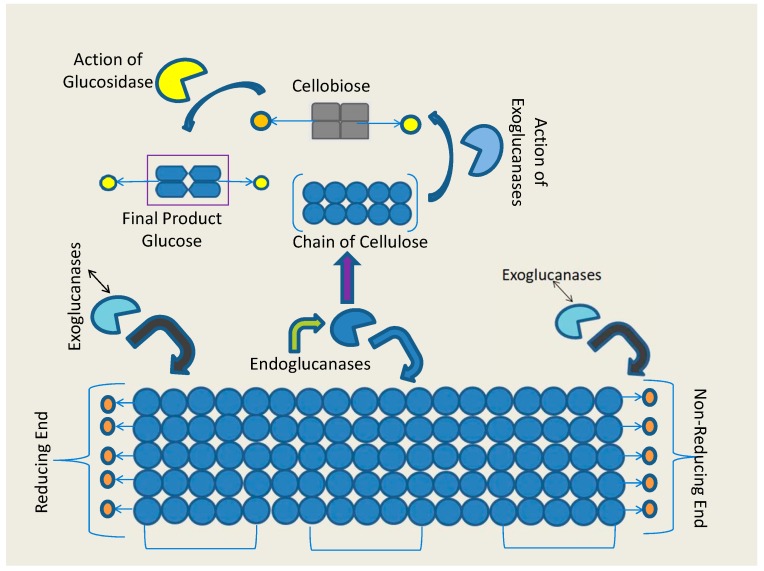
Hydrolysis of cellulose by the synergistic action of cellulases.

As discussed earlier, the β-glucosiadses discovered to date are either glucose tolerant or glucose stimulated, and are categorized in GH1 and GH3, respectively [52]. Very extensive studies were carried out by the Wither’s group [142,143] and others, for revealing the topology of the active site and for describing the catalytic mechanism of β-glucosidase. Figure 2 illustrates the generalized action of β-glucosidases, along with the other enzyme components of cellulase.

Various methods have been used to reveal the catalytic mechanism of the enzymes belonging to family GH1, for example, dependence on pH, inhibition, effect of deuterium isotopes and structure reactivity [142], flurosugar labeled essential amino acid [143], reactions with analogues of deoxy substrates [144], and site directed mutagenesis [145]. Members of the GH1 (glycosyl hydrolases 1) family retain the anomeric carbon configuration of the substrate, while catalyzing their hydrolysis [146]. The catalytic process of β-glucosidase involves two particular steps of glycosylation and deglycosylation, and the double displacement of two –COOH groups present at the active site of the enzyme, which function as nucleophile (conserved ‘I/ VTENG’ motif) and general acid/base (conserved “TFNEP” motif) [52,146,147].

**Figure 3:**
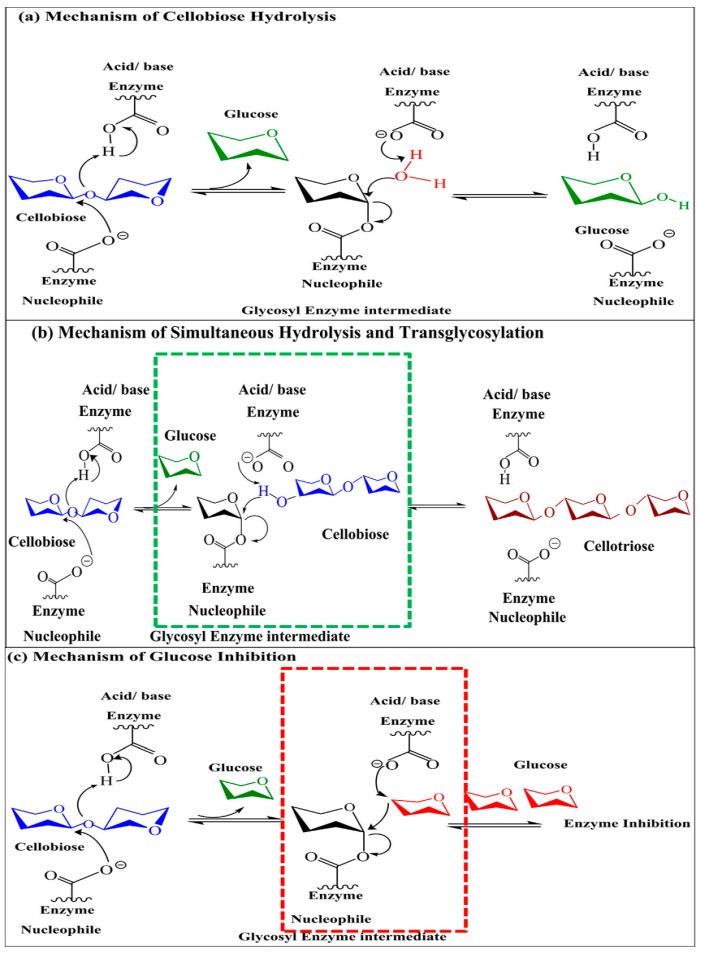
Schematic representation of the β-glucosidase mechanism of action: (**a**) hydrolysis, (**b**) simultaneous hydrolysis and transglycosylation, and (**c**) glucose inhibition. The transient glycosyl enzyme intermediate (E-Glu1*) is represented in the green box, while the glucose inhibited state (E-Glu) is represented in the red box (adopted with permission from the authors of [151], and also credit to [153]).

In the step of glycosylation performed by β-glucosidase, one –COOH group of the active site acts as a nucleophile to perform nucleophilic attacks on the anomeric carbon, forming a glucose–enzyme intermediate [148]. The other carboxylic group acts as a proton donor (acid), which cleaves the glycosidic bond by donating H^+^ to the oxygen of the O-glycosidic bond, and that leads to the glycosil–enzyme intermediate formation, and liberates aglycone [42,52]. The second step of deglycosylation involves the activity of the –COOH group acting as a base, which acted as a proton donor previously and now accepts the proton form a nucleophile such as water, monosaccharide, disaccharide, and monoterpenealcohol. It breaks the intermediate complex of the glucose–enzyme and sets the enzyme free with the production of a second product, and this completes the catalytic mechanism [149,150]. The catalytic mechanism of β-glucosidase of the family GH9 involves a single step, unlike the families GH1 and GH3 [141]. The β-glucosidases belonging to the GH9 family has an invert mechanism, which includes the nucleophilic attack of activated water on the anomeric carbon, which results in the single step displacement of the aglycone [141]. The displaced aglycone group gets protonated through the catalytic acid (proton donor), while a proton is extracted through the catalytic base (proton acceptor) from the water molecule [52,151] (Figure 3).

In a recent study, Dadheech et al. [152] performed the molecular docking of various β-glucosidase (AtBgl 1.1, 1.3 AtBgl 3.1, AtBgl 5.4, etc.) produced from *A. terreus* P14_T3 with the substrate cellobiose, in order to reveal the insight of the substrate hydrolysis. This study revealed that Glu, Asp, Trp, His, Tyr, and Arg, which are conserved amino acids, participate in the substrate hydrolysis, whereas Ser, Phe, Asn, and Gln take part in the formation of hydrogen bond and catalysis.

The possible mechanism of hydrolysis along with transglycosylation has been described in Figure 3. The –OH group of cellobiose performs a nucleophilic attack on the enzyme–glycosyl intermediate, and delivers the cellotriose and glucose with the (E-Glu1) transient glycosyl enzyme intermediate [131]. This nucleophilic attack ultimately results in the hydrolysis and transglycosylation reactions to occur simultaneously [151].

## 5. Inhibition of Enzymes during Saccharification

Several pretreatment techniques, like acid hydrolysis, alkaline treatment, steam explosion, hydrothermal processing, chemical treatment, and biological pretreatment, are being used to reduce the complexity and recalcitrance of the lignocellulosic biomass [154]. Although these pretreatment methods make cellulose more available to cellulolytic enzymes, on other hand, they also lead to the formation of undesired byproducts of lignocelluloses. These included mainly (i) furan aldehydes, such as furfural and 5-hydroxymethylfurfural; (ii) organic acids, like formic acid, acetic acid, and levulinic acid; (iii) phenols, such as gallic acid, hydroxynamic acid, and tannins [155]; and (iv)soluble mono- and oligo-saccharides [156]. The chemical composition and solid concentration of raw materials and the methods of pretreatment used are mainly responsible for the generation of the above-mentioned byproducts [154,157,158]. Byproducts derived from the pretreatment of the lignocellulosic biomass binds to the enzyme, hydrolyzing the cellulose and hence hindering the activity of the celluloytic enzymes up to a very high extent [159,160]. Soluble carbohydrates, aromatic compounds, and monosaccharides, like glucose and, recently, oligosaccharides liberated from xylan and mannans, have also been described to show inhibitory effects on the catalytic efficiency of enzymes [64,161].

In order to overcome the drawbacks of enzyme inhibition, several strategies have been proposed that may be helpful to minimize the inhibition of enzymes. The recalcitrance of any lignocellulosic substrate is the major reason that necessitates the pretreatment. However, the feedstocks having less recalcitrance, such as the tree of *Populus trichocarpa*, may be chosen as a suitable biomass for biofuel production [162]. The most powerful and generally most used method for minimizing the inhibition problem is the detoxification of the lignocellulosic hydrolysates and slurries with the help of chemical additives like a reducing agent, alkali, and polymers [163,164]. Other techniques like liquid–liquid and liquid–solid extraction, like ion exchange [165], heating, and vaporization, and enzymatic treatments may also be used [158]. Bioabatement can also be opted for in the process of detoxification, which includes treatment with microbes and hence facilitates the hydrolysis of cellulose through enzymes and improves the fermentability of cellulose [166,167]. Apart from bioabatement, modifying the culturing scheme may also help in minimizing the inhibition of enzymes, such as by using consolidated bioprocessing, where simultaneous cellulosic hydrolysis and microbial fermentation may check the product inhibition [154]. Apart from these strategies, some other methods may also be opted for, such as selecting the microorganism with potential for resistance to inhibitors [168]. The evolutionary engineering of the microorganism [169], and genetic and metabolic engineering may also provide the desired results [170]. Hence, these strategies may be helpful to tackle the problem of the inhibition of enzymes.

## 6. Challenges and Future Prospects

As discussed in the above sections, β-glucosidase is a key factor in the bioconversion of biomass to biofuel, but because of the poor yield, lack of a potential BGL producing microorganism, and low activity, this process still remains a bottleneck for industrial uses [57,171]. Several factors are challenging in biofuel production through cellulosic biomass, including β-glucosidase itself, such as the cost of the enzyme; the quantity and efficiency of the BGL [20];the inhibition of BGL through glucose, which blocks the binding of substrate to the active site of β-glucosidase [42]; and inhibition through other biofuel products. It was observed by Rajashree et al. that β-glucosidase isomers with a low molecular weight were more glucose tolerant, and the introduction of lactose into the media also increases the glucose tolerance [172].

In general, the selections of the best microorganisms, which are employed in the production of β-glucosidases, is the first step of cost cutting in the commercial production of β-glucosidase [33]. In a recent study, Gao et al. worked on *Penicillium piceum*, a strain with a hyper production of β-glucosidase, and further enhanced the activity of BGL by dimethyl sulfate (DMS) mutagenesis upto 53.12 IU/mL [110]. In another study, Garcia et al. used wheat bran as a substrate in the solid-state fermentation (SSF) for the production of β-glucosidase from *Lichtheimia ramose*, which resulted in a cost effective BGL production, because of the enhanced BGL production and cheaper substrate [173]. I-Son et al. worked with newly isolated *Penicillium citrinum*YS40-5, and the BGL produced by this strain was recorded to have a specific activity of 159.1 U/g [174]. They further described that using rice bran with added urea as a substrate under SSF condition was an economical approach, and was comparable to commercial Novo-188. Moreover, the produced β-glucosidase was also able to retain its 95% catalytic activity towards rice bran, even after the prolonged storage of four weeks [175].

Along with the selection of an efficient microbial strain, the substrate used in their culture is also of much importance, because this is the essential component for the growth of microorganisms, like sources of carbon and nitrogen, inducers, and so on, which are obtained from the substrate [33]. The numerous substrates used for the production of β-glucosidase are summarized in Table 2.

**Figure 4:**
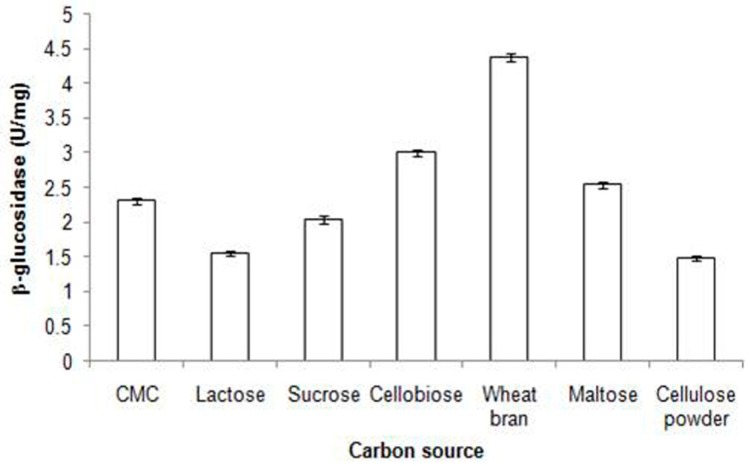
Different carbon sources on β-glucosidase production by *A. niger* NRRL 599 in shake flasks. ± indicates the standard deviation among the three parallel replicates. Incubation at 30 °C for 96 h at pH 5.5 in M-I medium (adopted from the literature [73]).

Nascimento et al. worked on *Humicola grisea* var. *thermoidea* for the production of β-glucosidase, and noticed that the highest yield of β-glucosidase was achieved when avicel (microcrystalline cellulose) was used as a substrate, but sugarcane bagasse was able to produce the BGL with the highest specific activity [184]. In a study on *Aspergillus sachharolyticus*, Sorensen et al. screened various carbon sources, ranging from simple monomers, such as xylose and polysachharide, like xylan, to complex substrates of lignocellulosic biomasses, such as corn cobs [185]. The findings of their study described wheat bran as the best substrate, giving highest β-glucosidase production and highest activity, and noticed that pretreated corn stover as a potential substrate. In a study, Zahoor et al. [73] worked on *A. niger* NRRL 599, and explored the effect of different carbon sources on the production of β-glucosidase (Figure 4) [73].

In a recent study on *A. oryzae*, Ghani et al. used rice bran and broken rice as a substrate in SSF, and also added soy bean waste as the nitrogen source [186]. These authors reported that the β-glucosidase produced using rice bran exhibited a higher activity (68.16 U/g) than the broken rice (3.96 U/g). Moreover, it was found that after the addition of 1% soy bean waste, the activity of BGL was increased to 2125.7 U/g. Hence, this study may be significant for cutting the cost of the β-glucosidase production. From the above-mentioned studies, it may be concluded that a perfectly optimized substrate, especially used in SSF, would be able to minimize the overall production cost of β-glucosidase.

Pryor and Nahar conducted an interesting study using response surface methodology (RSM), regarding the loadings of BGL and its effect on the hydrolysis rate of biomass. They used a dilute sulfuric acid pretreated corn stover as a substrate, and a BGL ranging from 0.5 to 2 CBU FPU^−1^. As per the experimental results, it was concluded that any loading of BGL above 0.2 CBU FPU^−1^ at an industrial scale had little practical effect on the hydrolysis while using the acid pretreated substrate [187]. However, these findings seem to be very lucrative from the point of view of the total cost of BGL, but this still needs advanced research. Moreover, for retaining the activity of the enzyme and the cost reduction of the process, enzyme recycling is also an effective approach [33]. Beta-glucosidase may also be reused by the immobilization, and hence the activity of BGL and the stability over various pH and temperature ranges can also be enhanced [188]. In a recent study by Moi et al., the β-glucosidase produced from *Thermoascus aurantiacus* was captured in anionic cryogel, which may function as a chromatographic media and as an immobilizer for β-glucosidase [189]. In a study by Chen et al., the immobilization of BGL was done on a nanoparticle of Fe_3_O_4_ with agarose, and after 15 consecutive cycles, ~90% of the enzymatic activity was recorded [190]. It was observed that the immobilized enzyme often ended up with an increased Km and decreased Vmax value, but their virtue of multiple time usage and their ability to withstand a wider range of pHs and temperatures makes the utilization of the immobilization process feasible [32,191]. In a recent study, Goffe and Ferrasse estimated the optimum efficiency for biomass to biofuel conversion on the basis of stoichiometry, which offers a new insight to evaluate the efficiency of the current bioconversion systems of the biomass. The methodology and proposed calculations may help one to find the optimal efficiency for the conversion of the biomass [192]. In one of the studies, Ahmed and Sarkar investigated the carbon emission effects on managing a sustainable supply chain system for the second-generation biofuel [193]. These studies may be helpful for developing and modifying the current understandings of the biofuel production. Moreover, these studies may help in economizing the commercial biofuel production technology. 

## 7. Conclusions

The conversion of lignocellulosic biomass to biofuel is a multistep process, and the second generation of biofuel production is still finding its way towards its commercialization. Numerous hurdles like the highly crystallized structure of cellulose, the costly step of pretreatment for reducing the crystallinity, and the delignification of cellulose impose great adversity in the commercialization of this technology. In addition, the high cost of enzymes and the absence of potential microorganisms for BGL production, as well as the slow enzymatic degradation, are the main obstacles, that are needed to be overcome during the enzymatic hydrolysis. This review discussed several methods for resolving the above-mentioned existing problems. A hyper β-glucosidase producer strain, the optimization of the substrate and other physiological parameters, and maintaining the efficiency of BGL and making it reusable through immobilization are various strategies. These strategies are likely to helpful in the development of the sustainable and economical production of BGL, and subsequently, its application in the biofuel production process.

## Figures and Tables

**Table 1:** Various microorganisms producing β-glucosidases under different physiological conditions and the activity of produced beta-glucosidase (BGL) in (IU/mL).

S.No	Microorganism	Physiological conditions(Temperature, pH, Mode of fermentation and substrate)				BGL Activity	Ref.
		Temp	pH	Mode of Fermentation	Substrate	(IU/mL)	
1.	*Trichodermaatroviridae* TUB F-1505 TUB F-1724 TUB F- 1753	30 °C 30 °C 30 °C	6.2 6.2 6.2	Submerged Submerged Submerged	Steam pretreated willow	5.30 11.70 10.28	[102]
2.	*Bacillus halodurans* C-125	45 °C	8.0	Submerged	Lactose induced Luria broth LB media	95	[103]
3.	*Aspergillusprotuberus*	30 °C	3.0	Solid state	Rice husk	26.06 IU/g	[104]
4.	*Pichiapastoris* Bgl gene from *Aspergillus niger*	30 °C	5.0	Fed batch	Glycerol+ methanol(1:5 ratio)	129	[105]
5.	*Candida peltata* NRRL Y-6888	50 °C	5.0	Submerged	Glucose+xylose+sucrose+ maltose+arabinose	1.5	[106]
6.	*Issatchenkiaorientalis*	50 °C	5.0	Submerged	Esculine	6 × 10^−3^	[107]
7.	*Bacillus licheniformis*	60 °C	7.0	Submerged	Glucose+ sucrose	45.44	[108]
8.	*Penicillium oxalicum*	30 °C	-	Submerged	Microcrystalline cellulose	150	[109]
9.	*Talaromycesamestolkial*	70 °C	4.0	Submerged	Glucose	1.8	[110]
10.	*Penicillium piceum*	55 °C	5.0	Submerged	Avicel	53.12	[111]
11.	*Penicilliume chinulatum*	50 °C	4.8	Submerged	Microcrystalline cellulose+glucose+ soy bran	1.5	[111]
12.	*Saccharophagus degradans* *, 2-40^T^*	30 °C	6.0	Submerged	Laminarin	-	[112]
13.	*Micrococcus antarcticus*	25 °C	6.5	Submerged	Cellobiose	289	[113]
14.	*Aspergillus awamori*	28 °C	4.5	Solid state	Pineapple crown leaves + wheat bran	820 ± 30 IU/g	[114]
15.	*Aspergillus awamori*2B.361 U2/1	30 °C	8.0	Submerged	Wheat bran	104.7	[115]
16.	*Penicilliumsp.* LMI01	60 °C	6.0	Submerged	Carboxymethyl cellulose	0.058 ± 0.004	[116]
17.	*Aspergillus niger and Aspergillus oryzae*	28–30 °C	-	Solid state	Sugarcane bagasse	814 IU/g	[117]
18.	*Aspergillus flavus*	37 °C	-	Submerged	Wheat bran	0.64	[118]
19.	*Aspergillus flavus* ITCC 7680	30 ± 2 °C	4.8	Solid state	Pretreated cotton stalk	96 ± 2.9 IU/g	[119]
20.	*Bacillus subtilis* *CCMA 0087.*	36.6 °C	3.64	Submerged	Coffee pulp	22.59	[120]
21.	*Lichtheimia ramosa*	32 °C	-	Submerged	Flaxseed	3.54	[121]

**Table 2:** Numerous studies on the production of β-glucosidase using different types of substrates.

S. No	Microorganism	Carbon Substrate	Activity of β-glucosidase (IU/g)	Reference
1.	*Aspergillus fumigatus*	Microcrystalline cellulose (Avicel) Kraft pulp	27.5 5.68	[175]
2.	*Penicillium verruculosum*	Alkali pretreated passion fruit peel	8.54 IU/ml	[176]
3.	*Lichtheimia ramose*	Wheat bran Soy bran Sugarcane bagasse	162.2 ± 4.2 11.5 ± 0.7 11.1 ± 0.25	[173]
4.	*Aspergillus niger*SCBM1	Biomass sorghum +0.5% peptone	54.90	[177]
5.	*Aspergillus ibericus*	Washed seaweed	6.94 ± 0.21	[178]
6.	*Pleurotus pulmonarius*	Spent mushroom	6.83	[179]
7.	*Byssochlamys spectabilis* *Lichtheimia corymbifera*	Wheat bran Wheat bran	51.0 ± 0.75 11.6 ± 0.8	[180]
8.	*Bacillus subtilis* PS-CM5-UM3	Citrus sinensis bagasse +1% peptone	264.0	[181]
9.	*Gongronella butleri*	Wheat bran	215.4	[182]
10.	*Penicillium oxalicum*GZ-2	Rice straw	2.7 IU/mL	[183]
